# Sharing Research Data to Improve Public Health: A Funder Perspective

**DOI:** 10.1177/1556264615593485

**Published:** 2015-07

**Authors:** David Carr, Katherine Littler

**Affiliations:** 1 The Wellcome Trust, London, UK

**Keywords:** biomedical research ethics, data sharing, data release, research data, research governance, low income countries, clinical research, health policy

## Abstract

Through the Public Health Research Data Forum, global health research funders are working together to increase the availability of public health and epidemiology research data in ways that are equitable, ethical, and efficient. The Wellcome Trust funded the research reported in this special edition as a first step toward building an evidence base on the perspectives of research stakeholders in low- and middle-income countries on the benefits and challenges of sharing health research data. We hope this work will make a key contribution to discussions aimed at creating policy frameworks for data access at local, national, and regional levels that are sensitive to different contexts and ensure the benefits to research and health are realized in an equitable manner.

As a charitable research foundation, the Wellcome Trust is strongly committed to ensuring that the outputs of the research we support—including research publications and data—can be accessed and used in a manner that delivers the greatest possible benefit to global health. In common with other research funders around the world, we believe that many of the increasingly rich and complex datasets that are generated by the research we support have to date been largely untapped resources of considerable value. Through making these data more widely accessible and useable to other researchers, they can be used to advance avenues of research that were never envisaged by the original data generator, as well as to verify published research findings.

In this light, many funders have introduced explicit policies on data management and sharing that require the researchers we support to maximize access to datasets in a timely and appropriate manner and with as few restrictions as possible (Wykstra, 2013). In implementing their policies, funders are aware that there are significant challenges to overcome. Different research disciplines are at very different stages in terms of developing the infrastructures, resources, and skillsets needed to manage and share data. Some sections of the research community have been much quicker to embed data sharing practices than others, and across the research enterprise, incentive structures do not yet recognize adequately the cost and effort required to generate and make available high quality data resources. Furthermore, for many areas of biomedical research that involve the collection of data from patients and populations, limits on data sharing are required to safeguard the privacy of research participants.

Public health and epidemiology research has been highlighted as an area in which tremendous benefits could be gained from data sharing, but where the community has been relatively slow to embrace it and where key enabling infrastructures are not yet in place (Pisani & AbouZahr, 2010). This is in part the result of some justifiable barriers and concerns (van Panhuis et al., 2014). In particular, researchers who invest significant time and effort in compiling large-scale long-term datasets from cohorts and populations are understandably reluctant to share these data before they have had sufficient time to conduct and publish their own analyses. This concern is amplified for researchers in resource-poor settings, where fears are particularly acute that the benefits will be lost to better resourced groups with enhanced capacity for data analysis. There may also be particular ethical risks to individuals and populations in these settings who may be at heightened risk of stigmatization or harm if their privacy is compromised.

While recognizing the need to address these concerns, we would argue that there is an “ethical imperative” to support data sharing to ensure that datasets collected at considerable expense using public and charitable research funds are used in a manner that achieves the greatest possible benefit to health and society. Following a lengthy period of discussion and debate, a group of 17 global health research funders from across the world came together in 2011 to publish a joint statement of purpose on sharing research data to improve public health (Walport & Brest, 2011). The statement articulated a shared vision to increase the availability of health research data in ways that are equitable, ethical, and efficient and will accelerate improvements in public health.

To advance this vision, the partner funders established the Public Health Research Data Forum (www.wellcome.ac.uk/PHRDF) as a mechanism to help coordinate funder activities and develop joint activities to further the goals of the joint statement. Over the last 3 years, the Forum has commissioned several major projects to investigate key issues and challenges associated with increasing the availability and use of health research data—publishing reports on data citation (Rans, Day, Duke, & Ball, 2013), enhancing data discoverability (Public Health Research Data Forum, 2014), and enabling data linkage (Public Health Research Data Forum, 2015).

Forum partners have been aware from the outset that it will be vital to build and maintain the trust and support of all the stakeholders involved in the research process. These include researchers who generate and wish to use data, research institutions, local ethical review bodies, and critically, the research participants and populations who are making their data available for research. We are also very mindful that the policy and governance frameworks that are required to achieve this may be highly context-dependent—recognizing that the way our policy requirements are implemented in practice needs to be adapted in line with the varying values and cultural norms that exist in different settings across the world.

From an early stage in the Forum’s existence, it became clear that while we had some evidence on the views of stakeholders in high-income settings on the use of personal data in research, next to nothing was known about the perspectives and expectations of research participants and stakeholders in low- and middle-income country (LMIC) settings. On behalf of the Forum partners, the Wellcome Trust commissioned the research reported in this special edition to target this critical evidence gap—linking the expertise of the Ethox team at the University of Oxford with research studies in Kenya, India, South Africa, Thailand, and Vietnam. A key objective of the project from the outset was to produce a toolkit that would enable the methodologies and resources developed through the project to be applied and built upon in other settings.

The findings reported in the articles published here cast valuable new light on the views of LMIC stakeholders on the opportunities and challenges associated with data sharing, revealing not only some common themes between the five study sites but also some important differences. To mark the culmination of the project, the Forum partners held a workshop in Cape Town, South Africa on March 29 to 31, 2015, to discuss the preliminary findings of this work with key African research stakeholders, and consider the key opportunities and challenges for advancing data sharing in an African context.

Discussions in Cape Town emphasized the need for data sharing to be underpinned by fair and equitable terms, which ensure benefits are delivered to local communities and that local capacity to analyze and use data is enhanced. There is a strong preference from LMIC researchers for data to be shared (at least in the first instance) in a context of established and trusted collaborations and networks: Concerns are amplified significantly when stakeholders are asked to consider sharing data to users with whom these links do not exist.

Second, protecting the privacy and confidentiality of research participants is clearly of paramount concern. A key challenge in establishing effective governance frameworks is balancing the need to ensure that data can be shared for beneficial purposes with the need to create proportionate safeguards to guard against these risks. A related challenge is the need to understand and develop the governance mechanisms that are necessary to underpin and legitimize broad consent models that allow for data to be used for beneficial research purposes that may currently be unforeseen.

Finally, there was a clear consensus that the importance of building trust and trustworthiness in frameworks established to enable data sharing cannot be over-emphasized. There is a need to actively engage key stakeholders in an ongoing dialogue around the benefits and risks, and to establish transparent and accountable data governance processes.

The outcomes of this project will play a major role in helping to shape the future priorities of the Public Health Research Data Forum. Moving forward, it is likely that the focus of the Forum will transition from commissioning research toward the development of resources, training materials, and technical support for researchers and institutions that aid them in establishing the infrastructures and policy frameworks needed to enable data sharing in different settings. The toolkit created through this research is the first major resource of this type.

We hope that this research will also make a broader contribution to discussions on the interplay of funder policies with appropriate local and national polices, and specifically how implementation of funder policies can be adapted locally to take account of contextual differences. It is also absolutely clear that the issues around data sharing are inextricably linked with broader issues in global health research—such as capacity building and benefit sharing—and a holistic perspective is required. Building on the discussions at Cape Town, the Forum partners are committed to facilitating an inclusive and wide ranging discussion on these issues—with the ultimate goal of ensuring that the benefits of data sharing are realized at both a global and a local level.
